# Active Inference and Auditory Hallucinations

**DOI:** 10.1162/cpsy_a_00022

**Published:** 2018-12

**Authors:** David Benrimoh, Thomas Parr, Peter Vincent, Rick A. Adams, Karl Friston

**Affiliations:** Wellcome Trust Centre for Neuroimaging, Institute of Neurology, University College London, London, UK; Wellcome Trust Centre for Neuroimaging, Institute of Neurology, University College London, London, UK; Wellcome Trust Centre for Neuroimaging, Institute of Neurology, University College London, London, UK; Division of Psychiatry, University College London, London, UK; Institute of Cognitive Neuroscience, University College London, London, UK; Wellcome Trust Centre for Neuroimaging, Institute of Neurology, University College London, London, UK

**Keywords:** Bayesian, active inference, schizophrenia, psychosis, hallucinations

## Abstract

Auditory verbal hallucinations (AVH) are often distressing symptoms of several neuropsychiatric conditions, including schizophrenia. Using a Markov decision process formulation of active inference, we develop a novel model of AVH as false (positive) inference. Active inference treats perception as a process of hypothesis testing, in which sensory data are used to disambiguate between alternative hypotheses about the world. Crucially, this depends upon a delicate balance between prior beliefs about unobserved (hidden) variables and the sensations they cause. A false inference that a voice is present, even in the absence of auditory sensations, suggests that prior beliefs dominate perceptual inference. Here we consider the computational mechanisms that could cause this imbalance in perception. Through simulation, we show that the content of (and confidence in) prior beliefs depends on beliefs about policies (here sequences of listening and talking) and on beliefs about the reliability of sensory data. We demonstrate several ways in which hallucinatory percepts could occur when an agent expects to hear a voice in the presence of imprecise sensory data. This model expresses, in formal terms, alternative computational mechanisms that underwrite AVH and, speculatively, can be mapped onto neurobiological changes associated with schizophrenia. The interaction of action and perception is important in modeling AVH, given that speech is a fundamentally enactive and interactive process—and that hallucinators often actively engage with their voices.

## INTRODUCTION

The phenomenology of auditory verbal hallucinations (AVH) is rich and heterogeneous (McCarthy-Jones et al., 2014), but at its simplest, it involves the perception of a voice in the absence of (verbal) auditory data. Bayesian theories of brain function (Friston, 2010) provide a way to operationalize this notion. Hallucinations can be conceptualized, in Bayesian terms, as representing false (positive) inference (Adams, Stephan, Brown, Frith, & Friston, 2013; Brown, Adams, Parees, Edwards, & Friston, 2013; Corlett & Fletcher, 2009; Powers, Mathys, & Corlett, 2017; Teufel, Fletcher, & Davis, 2010; Teufel et al., 2015). The inference that a voice is present in the absence of auditory input implies that internally generated “prior” beliefs about the presence of stimuli dominate perception, even in the absence of supportive sensory evidence. This suggests that overly precise (confident) prior beliefs (Friston, 2005) could be important in the genesis of schizophrenic hallucinations. A complementary perspective (Adams et al., 2013) is that false inference may be due to a down-weighting of the precision of sensations (i.e., silence) that contradict the expected percept (i.e., a voice). Both forms of imbalance lead to excessive confidence in prior beliefs relative to sensory evidence. A Bayesian approach to understanding hallucinations affords an opportunity for the construction of computational models of the phenomenon, which could shed light on information processing deficits in schizophrenia and suggest potential neural mechanisms for further investigation.

A growing body of empirical work has investigated whether an overcounting of prior beliefs relative to sensory evidence may underlie some psychotic phenomena. Vercammen and Aleman (2010) conducted a behavioral experiment in healthy volunteers in which subjects were presented with sentences whose final “word” was predictable and overlaid with white noise, less predictable and overlaid with white noise, or simply white noise. They found that “top-down” hallucinations—defined as hearing a word that was not present but was predicted by the semantic context—were positively correlated with participants’ hallucination proneness. Powers et al. (2017) found that subjects with hallucinations (with or without psychosis—the latter being healthy voice hearers) were both more likely than non–voice hearers to experience hallucinations engendered by Pavlovian conditioning. A Bayesian model of these data indicated that hallucinating subjects had increased weighting of perceptual beliefs relative to sensory evidence. Teufel et al. (2015) found that people in early psychosis and those high in psychosis-like traits made greater use of prior knowledge when making decisions about ambiguous two-tone images (here the prior knowledge was conferred by showing color versions of the images). Finally, Cassidy et al. (2018) have shown recently that there is an overweighting of prior expectations (in conditions of uncertainty) in unmedicated patients with schizophrenia in an auditory task—and that this was both correlated with striatal dopamine release and could be induced by administration of amphetamine. As such, there is accumulating evidence that an overweighting of prior beliefs relative to sensory evidence relates to AVH; what is less clear is what kinds of priors may underwrite the development of AVH (given that most of the priors in the aforementioned experiments were supplied by the investigators). Previous computational models—focusing on perception—have simulated hallucinatory percepts via computational “lesions,” leading to the dissociation between sensations (input information) and percepts (outputs) that underwrite hallucinations. Hoffman and McGlashan (2006) produced a neural network model of hallucinations. This network was designed to model speech perception and was found to hallucinate when the model’s “working memory” layer was disrupted by overly pruning the connections between artificial neurons (i.e., dissociating percepts and sensations). To translate this idea to a Bayesian setting, we must introduce the concept of a generative model. This is a probabilistic model that expresses the (subpersonal) beliefs that the brain holds about the way in which its sensations are generated. Sensations can be dissociated from percepts if there is low confidence or precision (high variance) ascribed to sensory evidence. This low precision renders posterior beliefs about the causes of sensations poorly constrained by sensory evidence. Adams et al. (2013) demonstrated the face validity of this notion through simulating birdsongs under a hierarchical predictive coding model. In their simulations, they showed that, when an expected sequence in a song is omitted, a reduction of the precision of the likelihood (probability of sensations given their causes) produced a false percept (hallucination). Crucially, both the Hoffman and Adams models contain sequences of words or sounds and produced hallucinations in the context of degraded perceptual processing.

These formal models serve as a foundation for more complete computational accounts of auditory hallucinations. However, neither takes the active nature of perception into account. In this article, we argue for the importance of action, noting that the experience of auditory hallucinations is often in the form of a dialog with a voice, in which the hallucinating person may take an active role (i.e., speaking to or attempting to ignore the voice). So, in addition to the degraded perceptual processing of sensory input, our model’s perceptual inferences also depend upon its beliefs about how it interacts with another (speaking) agent. In addition, we will use a discrete state formulation that is more consistent with the discrete nature of language (words, sentences, etc.) than the continuous formulations used in previous work. These two agendas (the prominence of action and the discrete formulation) are naturally modeled using a Markov decision process (MDP). This serves as the generative model for a synthetic subject who engages in active (Bayesian) inference (Mirza, Adams, Mathys, & Friston, 2016; Parr & Friston, 2017). We begin with a brief overview of active inference before specifying the generative model we have used and show how altering this model can lead to hallucinations. Note that here we explore only one possible computational alteration that could underpin AVH; we discuss some alternative mechanisms in the discussion. We conclude with a (speculative) discussion of the neurobiological plausibility of this model—and its implications for understanding the link between pathophysiology and psychopathology.

## MATERIALS AND METHODS

### Active Inference

Under active inference, agents use a generative model to infer the causes of their sensory experiences. Crucially, agents are equipped with the ability to act (e.g., sample their environment) to gather evidence for their beliefs about those causes. Formally, this means agents act to minimize their variational free energy (Friston, Kilner, & Harrison, 2006). Technically, free energy is a variational approximation to the surprise, surprisal, self-information, or the negative log (marginal) likelihood of an observation under an internal model of the world (Friston, 2012). Crucially, surprise or self-information is negative Bayesian model evidence. This means that self-evidencing is the same as minimizing variational free energy. The free energy can be written as (see Table 1 for a list of variables)

$$F=-E_{Q(\tilde{s})}[\ln P(õ,\tilde{s})-\ln Q(\tilde{s})].$$ (1)

Here *F* is the free energy, *õ* is the sequence of observations through time, *s* are unobserved or hidden states, and *Q* is an approximate probability distribution over *s*. *P* is the generative model that expresses beliefs about how sensory data are generated, that is, the co-occurrence of observations and hidden states. It is this that takes the form of a MDP.

**Table 1.** Variables used

**Variable**	**Description**
**A**^*a*^, **A**^*p*^	Likelihood matrix (superscript denotes auditory or proprioceptive)
**B**^*a*^, **B**^*p*^	Transition matrix (superscript denotes auditory or proprioceptive)
*o*_*τ*_^*a*^, *o*_*τ*_^*p*^	Outcomes (agent observations; superscript denotes auditory or proprioceptive)
*π*	Policies
*ζ*	Likelihood precision
*γ*	Prior precision over policies
*s*	Hidden state (superscripts can be used to denote modality and subscripts to denote parameterization)
**G**	Expected free energy
**F**	Free energy
**C**	Prior preferences matrix
**D**	Beliefs about initial state

#### Markov Decision Process and Generative Model

A MDP is a framework for modeling the beliefs of agents who, like us, navigate environments in which they have control over some variables but not over others. MDPs have two important types of hidden variables that need to be inferred by the agent: hidden *states* and hidden *policies*. By *hidden*, we mean those variables that cannot be directly observed. The hidden states *s*_*τ*_ represent the beliefs of an agent about the causes of her sensations; for our purposes, these are “speaking” (or not) and “listening to a voice present in the environment” (or not). The hidden states are inferred from the sensory outcomes *o*_*τ*_ (where *τ* indexes time): auditory input being present (or not) and speech movements (i.e., proprioception) being present (or not). Put simply, the hidden state of “listening to a voice” implies outcomes in the auditory domain only; the hidden state of “speaking” (note that the agent does not automatically “know” it is speaking: It must infer it) implies outcomes in the proprioceptive domain only. Given that our agent employs sensory attenuation, when speaking, it reduces the precision of the auditory modality, which may affect its inference about the auditory state of the world. This means that speaking involves attending away from the auditory domain (to attenuate any auditory evidence that one is in fact not speaking). Instead of influencing outcomes directly, speaking modulates the precision of outcomes, given the listening state.

The probability of a sensory observation, given a hidden state, can be expressed as a likelihood matrix with elements *P*(*o*_*τ*_ = *i*|*s*_*τ*_ = *j*) =A_*ij*_. For the proprioceptive modality in our model, this was simply an identity matrix (mapping speaking to proprioception). For the auditory modality, the likelihood matrix is

$${\overset{-}{A}}_{a}=\frac{1}{Z}{\left(I_{2}+\exp(-30)\right)}^{\zeta }.$$ (2)

Here *Z* is a normalizing constant (i.e., partition function) that ensures that each column in this matrix sums to 1. *ζ* is the likelihood precision.^1^ **I** indicates the identity matrix; exp(−30) denotes a small amount of imprecision (added to each element of the matrix to avoid numerical overflow). The bar notation means that **A**_*a*_ has been normalized (subscript denotes the auditory modality). As *ζ* increases, this mapping comes to resemble the identity matrix. As it decreases, the probabilities become close to uniform, and the mapping becomes more uncertain. In other words, even if one knows the hidden state of the world, all outcomes are equally likely.

**Figure 1.**
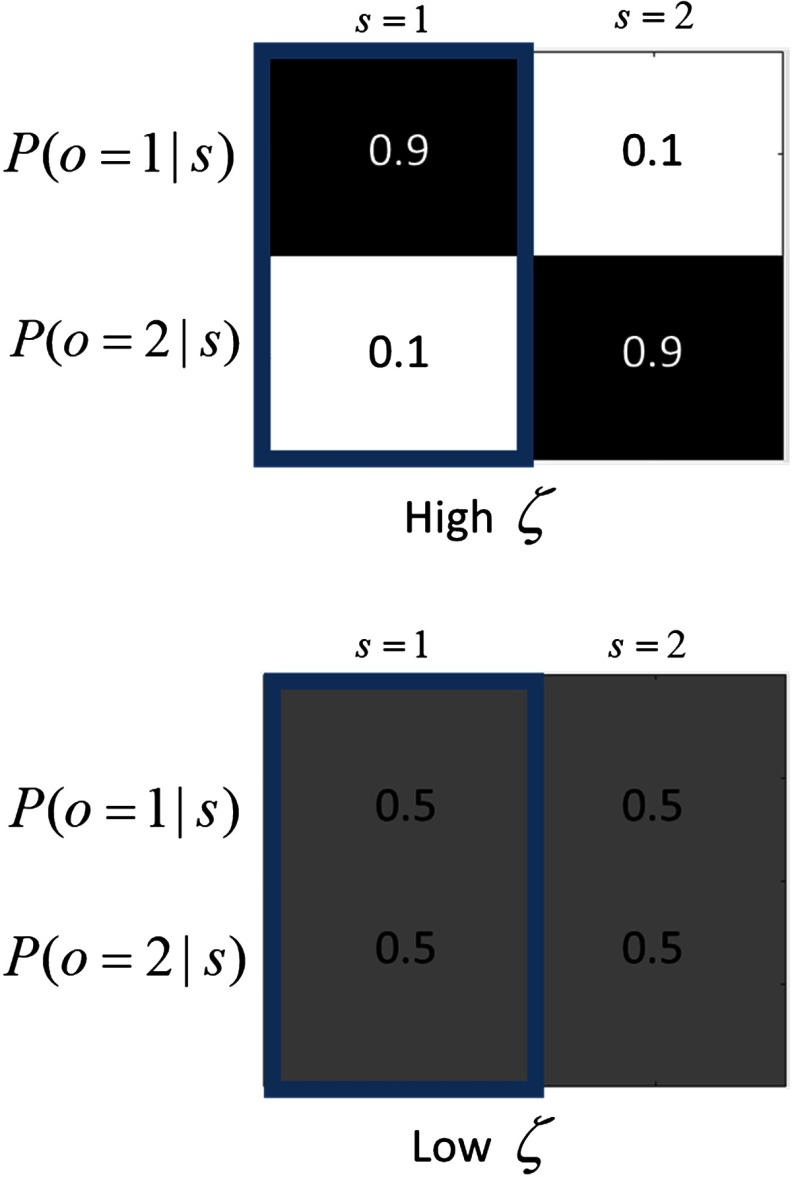
**Effect of likelihood precision on state–outcome mapping.** Top: A relatively precise likelihood matrix (high likelihood precision *ζ*) leads to a high-fidelity mapping between the state, *s*, and the outcome, *o* (in this case, if the state is equal to 1, then the probability of the outcome being equal to 1 is 0.9). Black = precise belief; gray = uncertainty regarding belief; white =4 very imprecise belief. Blue box highlights the probabilities of the outcomes associated with an arbitrary state. Bottom: In this case, the likelihood matrix has been made much less precise (all of its entries are now equal probabilities), corresponding to a low likelihood precision *ζ*. This leads to an uncertain state–outcome mapping.

Crucially, this formulation allows the fidelity of the mapping between states and outcomes to be modulated by *ζ* (see Figure 1 for a graphical illustration of this). In continuous state space formulations of active inference, optimizing the equivalent quantity is the process of attending, for example, attention to a sensory channel increases its precision (Feldman & Friston, 2010). Decreased likelihood precision is analogous to a reduction in signal to noise. Previously, we have shown that synthetic subjects tend to “ignore” low-precision mappings, as these contain relatively imprecise information (Parr & Friston, 2017; cf. the *streetlight effect*, Demirdjian et al., 2005).

**Figure 2.**
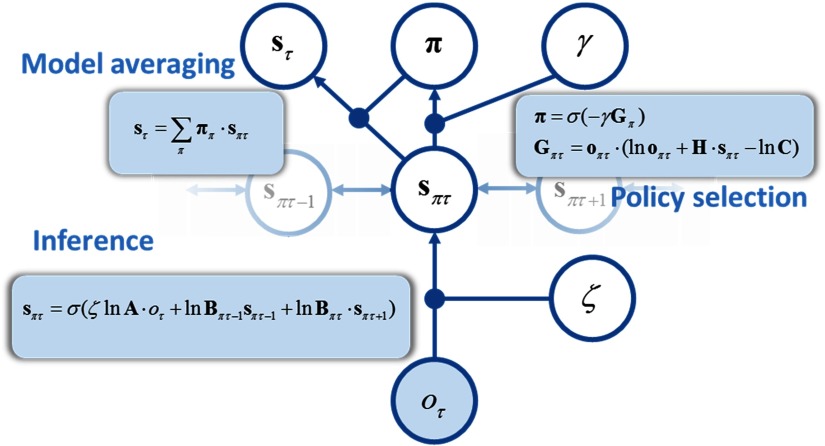
**Neuronal message passing.** This schematic illustrates the form of the (variational) message passing implied by active inference. Here sensory observations *o*_*τ*_ inform beliefs about states under each policy **s**_*πτ*_ (and this depends on the likelihood precision, *ζ*). These reciprocally influence beliefs about states in the past and future. Beliefs about states under each policy are used to compute the expected free energy for each policy. This informs the beliefs about policies, ***π***, and is modulated by precision over policies, *γ*. Beliefs about policies are combined with beliefs about the states under each policy to compute the marginal beliefs about states (averaged under all policies), **s**_*τ*_. By manipulating *γ*, *ζ*, and ***π***, we sought to induce changes in **s**_*τ*_. Bold terms represent vector quantities; italics are model parameters. **A** is the likelihood matrix; **B** is the state transition matrix; **C** is the prior preferences matrix. **G** is the expected free energy. **H** is the entropy of **A**. The filled circle containing *o*_*τ*_ is sensory data (outcomes).

When “I am speaking,” we set the auditory likelihood matrix to have equal probabilities for both listening and not listening to simulate “sensory attenuation.” This is the reduction of the precision afforded to self-generated sensory stimuli (i.e., the inability to tickle oneself; Blakemore, Wolpert, & Frith, 2000; Shergill, Bays, Frith, & Wolpert, 2003). Sensory attenuation is a fundamental aspect of intentional behavior, because it protects prior (intentional) beliefs about acting from sensory evidence that the act is not being executed (Brown, Adams, Parees, Edwards, & Friston, 2013). In effect, this means that while speaking, the presence and absence of sounds are deemed equally likely. This means I can maintain the belief that “I am speaking” in the absence of any auditory evidence to the contrary. Hence the agent’s inference about the current state depends only on prior beliefs. The complement of this is that, if an agent is listening and not speaking, it must deploy a higher level of likelihood (i.e., sensory) precision to “attend” to its conversational partner. In short, only when the agent is listening can the likelihood precision affect the mapping between sensations (outcomes) and percepts (states). See Equation 2 and Figures 2 and3.

**Figure 3.**
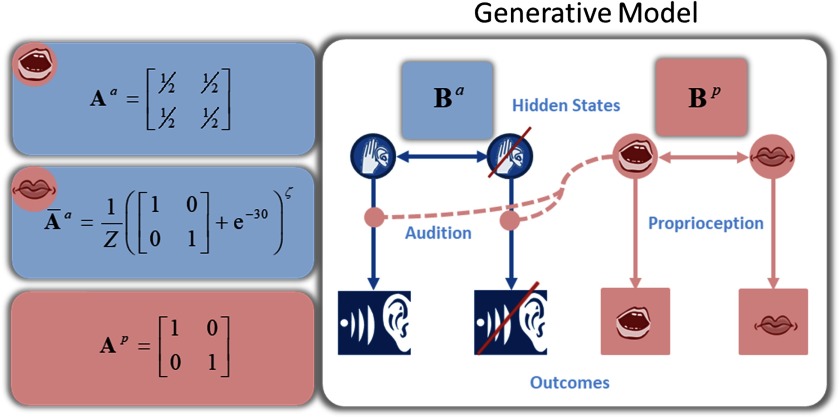
**Generative model.** Here the generative model is presented more explicitly. In blue are the hidden states for listening or not listening and their associated outcomes—hearing or not hearing a voice. Mapping between states and outcomes occurs via the likelihood matrix **A**. This is either the mapping from Equation 2 or (when the hidden state for speaking has been inferred) a matrix with equal entries to simulate sensory attenuation (the reduction of auditory likelihood precision during speaking, to prevent inference that one is not speaking but listening). In pink are the hidden states for speaking or not speaking, mapped via identity matrices to the speaking or not speaking proprioceptive outcomes. Transitions between hidden states are accomplished via the transition matrices **B**^*a*^ and **B**^*p*^.

The second type of hidden variable is the policy. Each policy is a sequence of actions that an agent can pursue. Crucially, the policy that the agent is currently pursuing must be *inferred* (Botvinick & Toussaint, 2012). Policies are simply various combinations of actions (e.g., listening or speaking) that, in our case, mimic the flow of a conversation. For example, the agent could take turns listening and speaking, could engage in a monologue, or could listen for the whole trial. This is closely related to formulations of active inference for birdsong, in which beliefs about the narrative of a given song are shared by two birds (Friston & Frith, 2015). In this article, the implicit “turn taking” is determined by the sequence of choices our agent pursues, as in real conversations.

The subject’s states change over time according to a probability transition matrix, conditioned upon the previous state and the policy, *π*, currently being pursued. This matrix is defined as B(*u*)_*ij*_ = *P*(*s*_*τ* +1_ = *i*|*s*_*τ*_ = *j*, *u* = *π*(*τ*)). In other words, the policy influences, via its effect on probability transitions, how a state at a given time step changes to become the state at the next. We constrained these policies so that deciding to listen requires one to stop talking, and vice versa. Heuristically, we consider listening as an action to be a composite of mental and physical actions: all the things one might do when expecting to hear someone speak (i.e., pay attention, turn your head to hear better, etc.; Holzman, 1972).

The subject was paired with a generative process that determined the sensory input she experienced: an environment that produces alternating sounds and silences. Whenever she chose to speak, this generated sound at the next time step (and attenuated the likelihood precision). Whenever the subject chose to listen, the likelihood precision was determined by Equation 2 at the next time step. In this way, our subject interacted with the (simple) generative process in the environment, generating sequences of sounds and silences dependent on both the environment and her actions.

The synthetic subject began the simulation with a probability distribution over possible initial hidden states, D_*i*_ = *P*(*s*_1_ = *i*). In our simulations, all initial states were equally likely. The subject is also equipped with a probability distribution over possible outcomes, which sets its *prior preferences*, C_*τi*_ = *P*(*o*_*τ*_ = *i*). These prior preferences influence policy choice by making some outcomes—and therefore the policies that tend to lead to those outcomes—more likely than others. In general, priors can be learned (empirical priors) or be “hardwired” into a phenotype by the pressures of natural selection (Friston, 2010). In this article, prior preferences did not differ between outcomes (i.e., there were flat priors over outcomes). This means that the imperatives for action (i.e., talking and listening) were driven purely by epistemic affordances, namely, the imperative to resolve uncertainty about states of affairs in the world (see later). Note that making the probability distributions over either initial states or preferred outcomes unequal did not affect the nature of the model’s hallucinations.

Importantly, there is also a prior probability distribution over policies. In this scheme, policies are treated as alternative models and are chosen via Bayesian model selection, where the policy selected leads to the lowest expected free energy, *G*(*π*). This is equivalent to saying that agents choose the policies that are most likely to resolve uncertainty (such as choosing to make a saccade to an informative location). Formally, one can express a prior belief over policies as

P(π)=σ(−γ⋅G(π)). (3)

Here the expected free energy under alternative policies is multiplied by a scalar *γ* and passed through a softmax function (i.e., normalized exponential) to return a prior distribution over policies. In this setting, *γ* is a sensitivity or inverse temperature parameter that signifies the precision, or *confidence*, the agent has about its beliefs about policies. How confident an agent is about its policies a priori will have an impact on the relative weighting of sensory information when the agent tries to infer its policy. The balance of likelihood and policy precisions will, in turn, determine whether sensory data—or the agent’s inferred policy—contribute most to the agent’s beliefs about states (i.e., listening to a voice or speaking). This policy precision is important in the current context, as midbrain dopamine has been suggested to be its in vivo homolog (Schwartenbeck, FitzGerald, Mathys, Dolan, & Friston, 2015). Before we can describe the form of expected free energy under each policy, we need to consider the form of the posterior beliefs.

To simulate active inference in a tractable manner, we adopt a mean-field approximation (Friston & Buzsaki, 2016) to update approximate posterior beliefs *Q* about hidden variables:

$$Q(\tilde{s},\pi )=Q(\pi )\underset{\tau }{\prod }Q(s_{\tau }|\pi ).$$ (4)

This formulation allows for independent optimization of each factor on the right-hand side of Equation 4 and for the expression of the free energy under a given policy as

$$F(\pi )=E_{Q}[\ln Q(\tilde{s}|\pi )-\ln P(õ,\tilde{s}|\pi )].$$ (5)

Free energy scores the information gained via observation. This poses a problem for policy selection: Policies should be chosen to reduce free energy in the future, but one can only define the free energy with respect to the present or the past (i.e., the times for which the agent has access to observations). To remedy this, we use the free energy expected under the policy to guide policy selection:

G(π)=EQ~[lnQ(s~|π)−lnP(õ,s~|π)],Q~(õ,s~|π)=Q(s~|π)P(õ|s~),P(π)=σ(−G(π)). (6)

**Figure 4.**
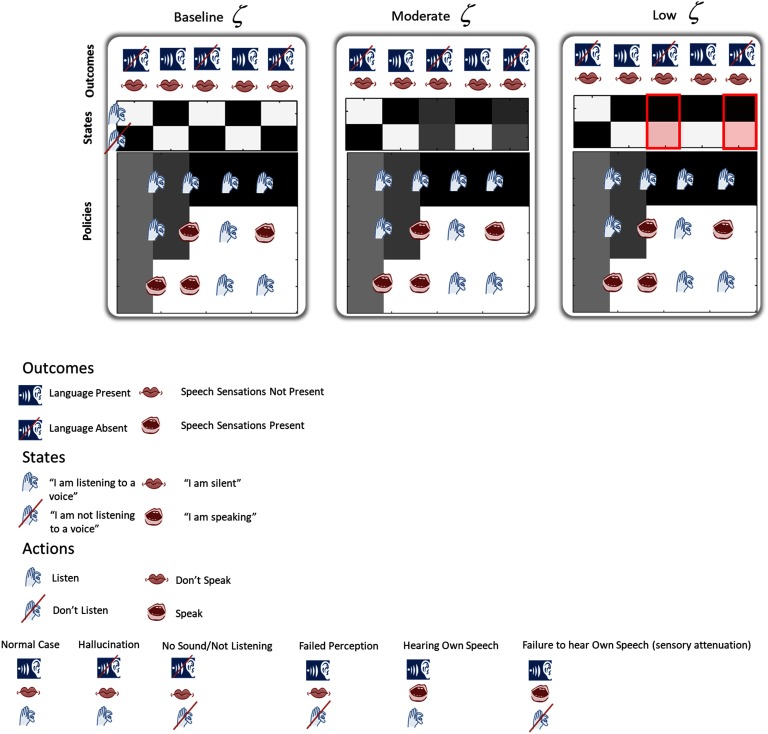
**An imprecise likelihood matrix can cause false positive inference.** The plots shown in this figure illustrate posterior beliefs about hidden states. True outcomes are noted above each trial. Darker colors mean greater probabilities of each state. Here we show that decreasing likelihood precision *ζ* (from left to right, *ζ* = 0.7; 0.525; 0.3) can lead to a false inference about the state of the world. Note that all other parameters were unchanged across these three simulations. Here we are looking at the beliefs of the agent about whether or not it is listening. In this example, it should listen (black box) and not listen (white box) in an alternating pattern to match the true outcomes of sound and no sound, respectively. Note that as there is an identity likelihood mapping between states and outcomes for speaking, the proprioceptive outcomes also indicate what the agent has inferred (i.e., whether it believes it is or is not speaking). In the leftmost figure, the agent infers the state of the world correctly, as reflected in its very certain beliefs (black squares) about states that correspond to external reality. In the middle figure, we have decreased the precision of the likelihood matrix, leading to uncertainty about whether or not the agent is in a listening state at the third and fifth time points; this decreased certainty is represented by the gray coloring over both possible states. In the third figure, a further decrease in *ζ* has led the agent to believe firmly that it is in the “listening to a voice” state (dark boxes at the third and fifth time points). These are the hallucinations (red circles). For Figures 4–6, the lower part of each panel, labeled “policies,” represents the inferences (posterior probabilities), over time, by the agent about which policy she is pursuing. Darker shading represents increasing probabilities. Each row represents an alternative policy. Plotted between the columns are the actions (listening or speaking) that would be selected during the transition between the preceding and the next time step, if the policy in that row were to be followed (i.e., in this figure, if Policy 1 is selected at the end of Time Step 3, the agent would choose to listen during Time Step 4). Note that the legend of icons is conserved for all figures.

Having specified the generative model (see Figure 4)—and in particular the prior beliefs about policies—in terms of expected free energy, it is relatively straightforward to derive belief update equations that underwrite perception and action. These equations of up-to-date posterior beliefs *Q*($\tilde{s}$, *π*) in response to new observations provide a minimization of free energy. Crucially, the posterior belief computed at one time step becomes the (empirical) prior for the next. We have argued previously (e.g., de Vries & Friston, 2017) that these update equations (aka variational message passing) can be interpreted in terms of neuronal message passing. The architecture of this message passing implies a connectivity scheme that closely resembles the functional architecture of cortico-subcortical loops (Friston, Rosch, Parr, Price, & Bowman, 2017).

In the foregoing, posterior beliefs about states are conditioned upon the policy pursued (consistent with the interpretation of planning-as-model-selection). We can marginalize out this dependence on policies (through Bayesian model averaging) to obtain a belief about hidden states:

$$Q(s_{\tau })=\sum_{\pi }Q(s_{\tau }|\pi )Q(\pi ).$$ (7)

Equation 7 is crucial because it means that beliefs about states (to the left of the equation) depend upon beliefs about the policy being pursued (the terms on the right, which correspond to the approximate probability distribution over policies, and over states, given policies). This is a fundamental observation that further ties perception to action. This speaks to a quintessentially enactive aspect of hallucinations—which we wanted to understand through simulations.

### In Silico Hallucinations

Given the definition of hallucinations outlined earlier, how might we induce hallucinations in our model? Our aim is to dissociate inferred states or beliefs about the world from sensory constraints. Given that the mapping between states and outcomes depends on the likelihood matrix and its likelihood precision parameter *ζ*, we can hypothesize the following:

1.  Decreasing likelihood precision *ζ* will affect the mapping between states and outcomes, with reduced *ζ* leading directly to a disconnect between the inferred state and the outcome (Equation 2). This follows because decreasing *ζ* is equivalent to reducing the confidence in sensory information, impairing the use of sensory evidence to inform perceptual synthesis.
2.  Increasing the prior precision over policies *γ* will change the prior distribution over policies (Equation 3) and hence the posterior beliefs about policies *Q*(*π*) and, therefore, inferred states through model averaging (Equation 7). Put simply, an increased policy precision will bias perceptual inference away from sensory evidence and toward its current action.
3.  Changing the policy space will also change *Q*(*π*) and therefore affect the state inferred through model averaging (Equation 7). Removing one or more policies may change which policy is inferred to be most likely, which in turn will affect state estimation.

## RESULTS: SIMULATIONS

### Reducing Likelihood Precision Leads to False Positive Inferences

**Figure 5.**
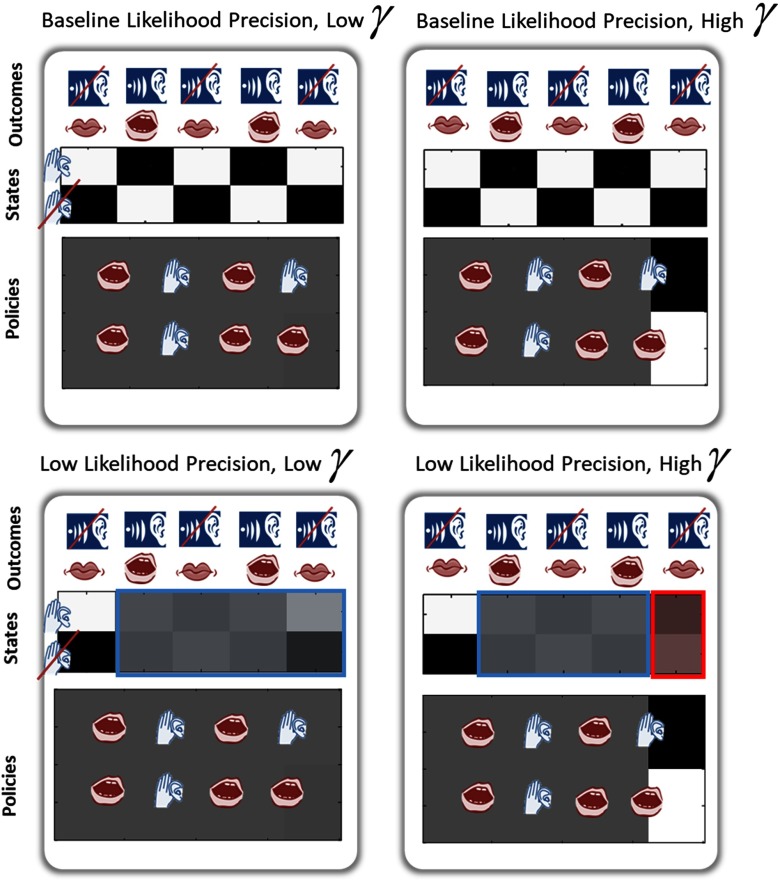
**Increasing prior precision over policies given a noisy likelihood matrix can induce hallucinations.** Darker boxes represent increasing levels of confidence. True outcomes are represented above each trial. On the left, an agent with a somewhat imprecise sensory mapping (likelihood precision *ζ* = 0.525) but a low prior precision over policies [*γ* = exp(64)] is uncertain about the state of the world and makes incorrect inferences (the inferred state does not concord with the outcome) at the second, third, and fourth time steps; at the third time step, the agent has hallucinated (infers it is listening to a voice when there is no voice present). Effects of low likelihood precision are denoted by the blue box. If *γ* is increased [*γ* = exp(−64)], the agent begins to hallucinate at the fifth time step as well (red rectangle). Note that this does not occur with baseline likelihood precision (*γ* = 0.7).

Inference about hidden states (e.g., “listening to a voice” or not) depends on both outcomes (whether sound is present) and policies (whether one is speaking or listening). We hypothesized that decreasing *ζ* could cause false inferences through a state–outcome dissociation. We additionally noted that, as inferences about states depend upon policies via Bayesian Model Averaging (see earlier), the inferred state will be influenced by the policy set. In Figure 5, we present the finding that reducing *ζ* moderately, from baseline, leads to perceptual “confusion” (i.e., posterior beliefs are ambiguous about which state is in play); further reducing the likelihood precision leads to a false positive inference, namely, the belief that the subject was listening in the absence of sound. The reason for this reversal is that the subject has inferred that it is following a policy consistent with the presence of heard speech. This induces an empirical prior belief that she is listening to something, despite the fact that there is nothing to hear, leading the agent to infer that she is actually hearing a sound in the environment. In the high-precision condition, this belief is corrected by precise sensory evidence—even if the agent chooses to listen, she should be able to infer when silence is present and conclude that she is not actually listening to anything in the environment. Low precision allows the (false) prior belief to dominate, producing a false inference. This illustrates that low precision has a permissive effect on hallucinations. As we will see in the third result, this effect of reduced likelihood (i.e., sensory) precision is highly dependent on the policy space; reduction of likelihood precision does not lead to hallucinations in *all* policy spaces.

### Increasing Prior Precision over Policies Can Elicit Hallucinations in the Presence of Reduced Likelihood Precision

We next demonstrate that increasing policy precision induces hallucinations in the presence of a permissive imprecise likelihood mapping between states and outcomes *ζ* and a suitable policy space. This effect is shown in Figure 5. Given normal *γ*, a lower *ζ* leads to some confusion at the last time step, but there is a weak (unconfident) belief in the not listening state, and as such, the agent concludes, accurately, that it has not heard a voice. Increasing *γ* then causes a switch to the (inaccurate) belief that a voice was heard.

Only a few policy spaces showed this effect of changing *γ*. This demonstrates an important finding: The generation of hallucinations is highly context dependent. More formally, the different policy options determine which policy is ultimately inferred. The most probable policy under the resulting approximate posterior distribution *Q*(*π*) has sensory consequences that might be consistent with, or may conflict with, the sensory evidence. False inference is induced by the latter. As such, hallucinations can only occur when the agent is equipped with a policy space likely to conflict with the sensory evidence (hence the policy space dependence of the effect of increasing *γ*). The degree to which the winning policy influences state expectations depends upon *γ*, which gives rise to more precise posteriors over policies. It is important to note again the permissive effect of unattenuated likelihood precision; at baseline *ζ*, increasing the prior precision over policies does not induce hallucinations. This is because conflict between policy and sensory evidence is more likely to be resolved in the winning policy’s favor if the influence of sensory evidence is down-weighted by a low precision.

### Hallucinations Are Context Dependent

**Figure 6.**
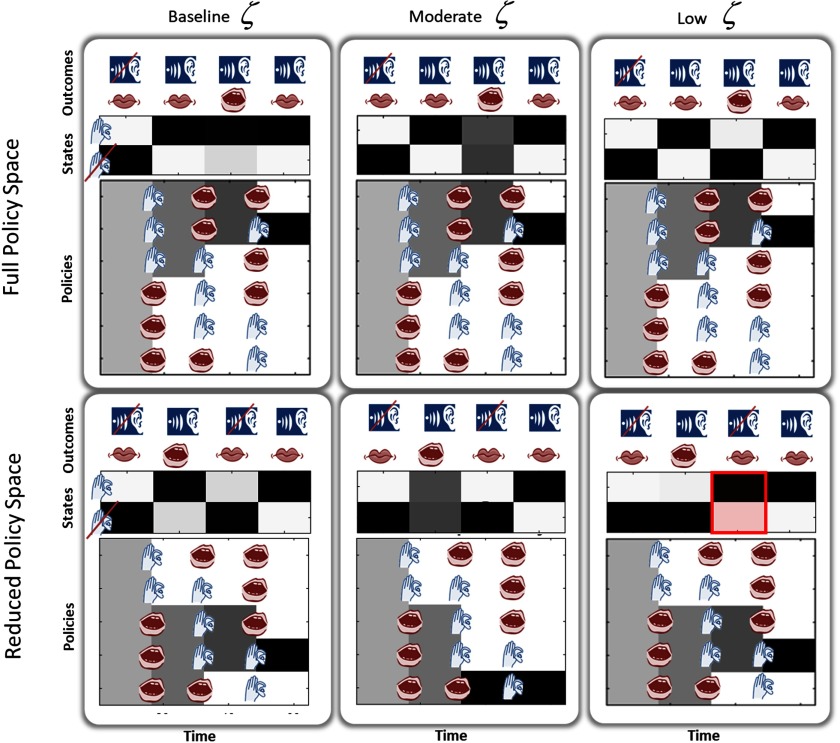
**Context sensitivity of synthetic hallucinations.** At high likelihood precision, outcomes dominate state inference; at low likelihood precision, priors derived from policies dominate and hallucinations can occur in a policy-dependent manner. This figure demonstrates that only certain policy spaces can lead to hallucinations, and only in the presence of permissively low likelihood precision. When the agent has a relatively large policy space (top row) and likelihood precision is reduced (left to right likelihood precision *ζ* = 0.7, 0.525, 0.3), the agent correctly infers the presence of externally generated speech. When likelihood precision is high (left), it believes that it is able to listen (third time step) while generating speech. This is inconsistent with the policies and indicates that precise sensory evidence dominates the empirical priors derived from policy selection. At a sufficiently low likelihood precision (right), beliefs about the policy dominate sensory evidence about states, and this causes our agent to believe that she was not listening, having selected the speak policy. This is permitted by the sensory attenuation we have associated with the speaking state and the oppositional nature of our policies (which contributed to the attenuation). On the plots of posterior probabilities of policies over time, we see that the agent has inferred that she is pursuing the second policy by the fourth time step. As such, the agent has concluded that this policy is the most likely given the data observed and the actions taken. If this policy is removed (bottom row), this agent now no longer has access to Policy 2 and has selected alternative policies. This poses no problem when likelihood precision is high (left), as sensory evidence again dominates the inference. When precision is low, however, this policy does not explain the data well, leading to a hallucination at the third time step (highlighted in red).

Figure 6 demonstrates the importance of the policy space in determining whether an agent will hallucinate. We equipped an agent with a set of six policies and decreased *ζ* in a stepwise manner, producing high-, moderately decreased, and low-precision conditions. With the full policy space (Figure 6, top row), the agent inferred a policy that induced no hallucinations. It did begin to stop hearing its own voice at one time at the lowest likelihood precision; this was enabled by the sensory attenuation associated with self-generated speech and by the way in which our policies are set up to be oppositional (which contributed to the attenuation). To illustrate the mechanisms by which certain policy spaces predispose to hallucinations, we “lesioned” the policy space by deleting the policy originally inferred by the agent (Figure 6, bottom row). This left policies that are worse explanations for active exchange with the same environment. The subject with the remaining five policies did not hallucinate at a high *ζ* but did hallucinate when *ζ* was lowered. This followed a dose–response relationship—at a moderately decreased *ζ*, the subject was confused about the state she was in but did not experience frank hallucinations. The lesioned policy space left the agent unable to appeal to the policy that better accounted for evidence in the auditory environment. When she was not able to use sensory information to correct her beliefs—about the actions to pursue (the low likelihood precision condition)—she was forced to infer a policy that led to a hallucination (Figure 6, bottom left). The subject hallucinated at the third time step because of the following sequence of events. At the second time step, she used the relatively precise information from the proprioceptive domain (which is not affected by the reduced likelihood precision in the auditory domain) to infer that she was speaking. Given that she was not speaking at the third time step, this left two plausible policies that allowed for speaking at the second and not speaking at the third time steps. Both of these mandated listening to a voice at the third time step, even though none was present. The subject was unable to use the imprecise auditory information to correct the prior engendered by the policy. The key point of these results is that different policy spaces are prone to different kinds of false inference under low likelihood precision—while false inference may occur in any policy space when the likelihood precision has been reduced, given the same environment and initial conditions, different policy spaces produce different kinds of false inference.

## DISCUSSION

We simulated a free energy–minimizing agent using a MDP formalism that engaged in a simple turn-taking conversation. We performed three in silico experiments on this synthetic subject to induce hallucinations (false positive inferences). We found that decreasing auditory precision, given a vulnerable policy space, could induce hallucinations, as could increasing the prior precision over policies in the presence of low likelihood precision. We found that the effect of both decreased likelihood precision and increased prior precision—over policies—was highly dependent on the policies available to the agent, with only a subset of policy spaces producing specifically false *positive* inference. Here we discuss our findings and relate them to possible underlying neurobiology.

### Precision and Prior Beliefs

Our first finding was that decreasing likelihood precision *ζ* can lead to hallucinations—in fact, no hallucinations in any of our simulations could occur without this deficit. *ζ* affects the (likelihood) mapping between states and outcomes. By attenuating the precision of this mapping, sensory evidence has less influence on belief updating; in other words, perception is dissociated from sensation. This is consistent with attractor and neural network models that produce hallucinations via similar means (Adams et al., 2013; Hoffman & McGlashan, 2006). As we will see shortly, the effects of a reduced *ζ* depend on the policy space; simply decreasing *ζ* does not always produce false *positive* inference.

Our second finding was that increasing the prior precision over policies also led to hallucinations, given reduced likelihood precision. This is because beliefs about policies (e.g., “now is my turn to listen”) are a source of prior beliefs about states (e.g., “I am hearing a voice”), which may be imposed on perception. This emphasizes the conflict between likelihood (i.e., sensory) and policy (i.e., prior) precision. An agent with strong (precise) prior beliefs about the actions it will take hallucinates because it is unable to correct for this prior belief using imprecise sensory evidence. An interesting consequence of this is that the form false inferences take becomes highly dependent on the action plans entertained.

Our third finding was that deleting the policy originally inferred by the agent to explain her actions (given the environment) leads her to hallucinate, given low likelihood precision; that is, the agent hallucinates when she does not have access to a model of the world that does not bias toward false positive inference and cannot use sensory information to correct false positive inferences under this poor model (note that the deleted “best” model of the world shown here does not necessarily result in perfect inference; it is only a *relatively* better model). Our model differs from previous models by incorporating actions specified by the policies employed by our simulated subject. As such, reduced likelihood precision is not sufficient to generate a hallucination (see Figure 6), and we were able to generate policy spaces that did not support false positive inference at low likelihood precision. Crucially, it is the interaction between likelihood precision and beliefs about the policy that then impacts the state the agent infers. This can be stated succinctly as follows: Agents will respond to reduced likelihood precision in a policy space–dependent manner.

The nature of this interaction is important. As in existing Bayesian accounts of hallucinations, our approach requires the imposition of (empirical) prior beliefs on perception. We propose a source for these empirical prior beliefs—beliefs about actions. By bringing in policies, we show how pathological empirical priors (over states) can develop from beliefs about “what would happen if I were to do that.” In short, we suggest that the balance between prior beliefs and sensory evidence can be framed as a balance between the confidence in plans of action and in the sensory consequences of these actions. An interesting consequence of this is that the form false inferences take becomes highly dependent on the plans selected.

Thus we arrive at a conceptualization of auditory hallucinations as requiring some defect in policy space (a policy space vulnerable to hallucinations, potentially with an increased prior precision) and decreased likelihood precision, which renders it impossible for sensory evidence to prevent the emergence of a false positive percept. This fits comfortably with Bayesian accounts of auditory hallucinations as resulting from prior beliefs about hearing a sound dominating over sensory evidence that a sound is not present.

Previous empirical work supports the hypothesis that both clinical and nonclinical positive symptoms are associated with greater reliance on priors relative to sensory evidence (Cassidy et al., 2018; Powers et al., 2017; Teufel et al., 2015), in particular, priors about voices in those who hear voices (Alderson-Day et al., 2017).

Conversely, many other phenomena in schizophrenia have been related to an increased weighting of sensory evidence relative to prior beliefs—the opposite imbalance (Adams et al., 2013). Reduced vulnerability to certain perceptual illusions, such as the hollow-mask illusion, is another key element of schizophrenia phenomenology and has been found in patients with positive symptoms (Notredame, Pins, Denève, & Jardri, 2014). Classically, perceptual illusions have been explained as obtaining when a prior belief (e.g., a bias toward detecting convex faces) dominates over sensory evidence (e.g., a concave face). This would seem to imply a weakening of priors in schizophrenia, allowing for a resistance to illusions. Indeed, Jardri and Denève (2013) used a belief propagation model (another Bayesian message-passing scheme) to show one way in which sensory evidence could come to dominate prior beliefs in the disorder. They proposed that a failure of interneurons to inhibit messages that have been passed up the hierarchy could lead to the content of those same messages being passed back down the hierarchy, in effect, mistaking sensory evidence for a prior belief and thus overweighting it. Likewise, an overweighting of sensory precision in a predictive coding hierarchy (as in Adams et al., 2013) would have the same effect. In addition to these purely perceptual tasks, an increased relative weighting of sensory evidence also accounts for sensorimotor (e.g., loss of smooth pursuit gain and failure to attenuate self-produced sensations; Shergill, Samson, Bays, Frith, & Wolpert, 2005), electrophysiological (e.g., diminished oddball responses), and belief-updating changes (Jardri & Denève, 2013) in schizophrenia. The apparent discrepancy between abnormally precise prior beliefs and a failure of sensory attenuation is resolved by noting that a failure to attenuate sensory precision does not preclude—and may even lead to—a relatively high prior precision (Adams et al., 2013). We now consider this in more detail.

How can these apparently conflicting findings be resolved? One potential explanation—developed in Sterzer et al. (2018)—notes that the cortical hierarchy is many levels deep and that prior beliefs in sensorimotor hierarchies are themselves contextualized by priors higher in the hierarchy. For example, the perceptual (predictive coding) hierarchies modeled by Adams et al. (2013), in which prior beliefs are relatively imprecise, essentially correspond to the likelihood matrix in the current model (i.e., perceiving a voice). Thus auditory sensations may be more vivid (increased sensory precision), but their content may be harder to resolve (decreased intermediate precision), and therefore they may be more easily dominated by beliefs about the speech narratives at a higher level. Indeed, increased sensory precision may give resulting hallucinations their realistic, “out-loud” quality.

The key element of both this and our previous modeling work is that for one to hallucinate, one’s prior beliefs must be unconstrained by incoming sensory evidence at some level of perceptual synthesis; that is, (empirical prior) precision must be reduced somewhere in the perceptual hierarchy. Our previous (predictive coding) model of birdsong could hallucinate only when sensory precision was reduced. While some auditory hallucinations are vague and ill formed, many have clear speech content, which in Bayesian terms must reflect priors of some sort. The model in this article is an initial attempt to show how beliefs about dialog with other agents are good candidates for such priors. Indeed, a propensity for dialog was the only attribute of inner speech associated with AVH proneness in a large population sample (McCarthy-Jones & Fernyhough, 2011).

Of course, there are numerous possible sources of priors for auditory hallucinations. Cluster analysis of phenomenological surveys indicates four different kinds (McCarthy-Jones et al., 2014): nonverbal, memories of a voice, one’s own voice, and another’s voice. So aside from prior beliefs about other agents, one’s memories and inner speech are also likely sources of priors in AVH, and in those with psychosis, delusional beliefs are likely to interact with these mechanisms. The idea that one’s own inner speech could contribute to AVH is long established and stems from the influential idea of a corollary discharge (i.e., descending predictions) failure in schizophrenia (Feinberg, 1978; Frith & Done, 1989). One difference between these accounts and current formulations—in terms of precision—is that in the case of motor passivity symptoms, an imprecise prediction is dominated by (precise) sensory evidence; in our AVH model, an imprecise likelihood is dominated by a prior belief. Clearly these failures of precision weighting may coexist in a deep hierarchy.

It is also realistic to suppose that delusional ideas may arise in circumstances of higher sensory precision (i.e., overly salient prediction errors causing unwarranted belief updates), but it is hard to think of fully formed delusions as anything but (high-level) prior beliefs held with undue precision. Delusions may also therefore arise from a similar pattern of altered precisions at different hierarchical levels. To address this empirically, it may be necessary to design tasks that can probe the precision beliefs at sensory, intermediate, and high hierarchical levels (Karvelis, Seitz, Lawrie, & Seriès, 2018).

Of course, other accounts could also be addressed. It is possible that precision imbalances are specific to different sensorimotor modalities—although work in the visual domain seems to argue against this (Schmack et al., 2013; Teufel et al., 2015). It is also possible that it is easier for subjects with schizophrenia to learn “empirical” perceptual priors over short timescales (e.g., if greater variance permits greater belief updating) but then harder to maintain their precision over time.

### Action and Planning

The current formulation distinguishes itself from previous models of AVH by incorporating action. In our model—and in active inference more generally—perception depends upon action, and what one perceives will depend on the balance between priors and the outcomes solicited by actions. As such, for a false positive inference to ensue, it is necessary for the policy that contributes to that inference to have a strong posterior probability, which is more likely to be the case if it has a greater prior probability. Both of our policy-related manipulations would have the effect of increasing this prior probability—this is caused directly by increasing prior precision over policies and indirectly by reducing the number of competing policies.

Our model’s use of action differentiates it somewhat from the “inner speech” models described earlier, which posit that hallucinations occur via the misattribution of self-generated internal speech as being generated by others (Allen, Aleman, & McGuire, 2007). In our model, hallucinations are *generated* to satisfy expectations of external speech derived from active perception. This generation is perhaps more in line with the phenomenology of AVH, which do not always take the form of one’s own thoughts that can be misattributed to another: They can have rich content that can take the form of conversations or that can have the grammatical structure of another’s speech (i.e., heard as “you” or “she/he” instead of “I”), and they can have their own personas (making them seem less like thoughts whose source was simply misattributed). That being said, as noted earlier, *dialogic* inner speech may certainly be one of the priors that drive AVH (McCarthy-Jones & Fernyhough, 2011). This may have implications for understanding the perceived loss of agency that often (but not always; McCarthy-Jones et al., 2014) accompanies AVH. We hope to explore this aspect in further work.

The inclusion of action, and the context of a dialog as opposed to the recognition of sequences of previously learned words, sets our model apart from Hoffman and McGlashan (2006). This is a key difference, because hallucinations can take the form of a conversational partner in schizophrenia. This indicates that the genesis and maintenance of hallucinations is unlikely to be solely sensory-perceptual in nature. The conversational aspect is naturally modeled using our approach, as it is a discrete time model, consistent with the sequential form of a conversation. A further attraction is that, as Dietrich and Markman (2003) argued, discrete representations are needed to allow for complex cognitive processes, such as categorization, which are likely relevant to psychotic phenomena.

### False Positives and False Negatives

Interestingly, Hoffman and McGlashan (2006) predicted—based on their simulations—that patients with hallucinations would fail to detect words at a higher rate than nonhallucinators. They confirmed this effect in a psychophysics study of hallucinating and nonhallucinating patients with schizophrenia-spectrum disorders as well as healthy controls. Furthermore, this effect was stronger when sensory stimuli were degraded (which can be regarded as an external modulation of the precision of the stimuli). Our model reproduces this latter effect—lower likelihood precision led to speech detection errors (i.e., inferring “not listening” when the true state was that sound was present) in several of our simulations.

### Neurobiological Plausibility

**Figure 7.**
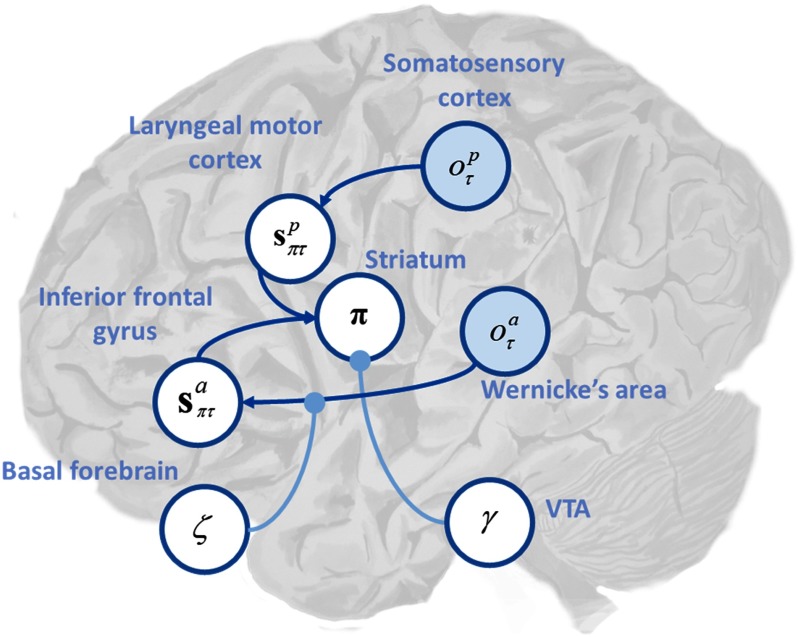
**Putative mapping of Markov decision process (MDP) model to neurobiology.** This figure shows the MDP model mapped onto putative neurobiology. This mapping should not be taken too seriously but serves as an illustration of how our model may relate to underlying functional anatomy. Here we have placed auditory outcomes, *o*_*a*_, in Wernicke’s area. Wernicke’s is connected via the arcuate fasciculus to the inferior frontal gyrus (IFG). The arcuate fasciculus represents the likelihood mapping between outcomes and auditory states, *s*_*a*_, which are located in IFG. Proprioceptive outcomes, *o*_*p*_, are located in primary somatosensory cortex and map to proprioceptive states (representation of whether or not one is speaking), *s*_*p*_, in laryngeal motor cortex (LMC). States in IFG and LMC inform the selection of policies assigned to the striatum; the blue lines represent corticostriatal connections between IFG and striatum and LMC and striatum. The nucleus basalis represents cholinergic signaling, and the likelihood precision *ζ* modulates (blue arrow) the state–outcome mapping in the auditory modality. The ventral tegmental area/substantia nigra (VTA) represents dopaminergic signaling, encodes the prior precision over policies *γ*, and modulates the striatum (light blue arrow).

Active inference can be formulated in terms of neurobiologically plausible processes (Friston, FitzGerald, Rigoli, Schwartenbeck, & Pezzulo, 2016). We can therefore draw some tentative parallels between the requisite neuronal computations and the pathophysiology that may underlie hallucinations. Figure 7 shows the MDP model mapped onto a putative functional architecture. Here we have represented auditory outcomes, *o*_*a*_, in Wernicke’s area. Wernicke’s area is a key region for the recognition of sequences of phonemes as constituting words (Ardila, Bernal, & Rosselli, 2016) and, as such, is a good candidate for one of the earliest processing centers in the brain capable of representing the presence or absence of meaningful speech. Wernicke’s area is connected via the arcuate fasciculus (which represents the likelihood mapping, **a**_*a*_, for the auditory modality) to the inferior frontal gyrus (IFG), where auditory states *s*_*a*_ may be inferred. IFG is an important part of the language network and has been suggested as a source of priors during speech recognition (Sohoglu, Peelle, Carlyon, & Davis, 2012) and the selection of semantic information (Grindrod, Bilenko, Myers, & Blumstein, 2008) and has been implicated in AVH in fMRI studies (Raij et al., 2009). In addition, a dynamic causal modeling study by Ćurčić-Blake et al. (2013) found reduced connectivity from Wernicke’s to Broca’s areas that correlated with patient AVH status, further supporting our model. Proprioceptive outcomes, *o*_*p*_, are assigned to primary somatosensory cortex and map via **a**_*p*_ to proprioceptive states, *s*_*p*_, in laryngeal motor cortex (LMC; Simonyan & Horwitz, 2011). Expected states in IFG and LMC inform the selection of policies, as performed by the striatum, which is well recognized to be involved in action planning. Under a neuronal process theory associated with active inference, probability distributions over policies are usually represented in the striatum, connected to expected states in the cortex via cortico-striato-thalamo-cortical loops (Friston, Rosch et al., 2017). Policy precision is often considered to be the computational homolog of dopamine (Schwartenbeck et al., 2015) and is represented here by the ventral tegmental area (VTA). Likelihood or sensory precision *ζ*, which may represent the computational homolog of acetylcholine (Dayan & Yu, 2001; Vossel et al., 2014), is located in the nucleus basalis (Liu, Chang, Pearce, & Gentleman, 2015).

Let us consider the potential neurobiological correlates of our modulation of *ζ*, *γ*, and *π*. Anticholinergic drugs like scopolamine can induce auditory hallucinations (Perry & Perry, 1995); muscarinic agonists have been shown, in small studies, to improve psychotic symptoms; and dysfunction of the muscarinic system is hypothesized to play a role in schizophrenia (for a review, see Raedler, Bymaster, Tandon, Copolov, & Dean, 2006). Thus there is some merit to the idea that the reduced *ζ* in our model may represent a cholinergic defect. However, the integrity of gray (Ohi et al., 2016) and white matter (including the arcuate fasciculus) is compromised in schizophrenia (Ćurčić-Blake et al., 2015; Gavrilescu et al., 2010), as is synaptic efficacy and NMDA receptor function (Coyle & Tsai, 2004). These abnormalities are also candidates for reduced likelihood precision, as they may represent a failure to propagate ascending sensory information.

In psychosis, there is increased presynaptic synthesis and release of striatal dopamine (see Howes & Kapur, 2009). This increase in striatal dopamine release may correspond to an increase in precision of policies *γ*, though this requires much more empirical validation.

Finally, what is the potential significance in neurobiological terms of our manipulation of the policy space? Policies could be encoded in the cortex, selected in the striatum through cortico-striatal loops, and accessed by other regions of cortex via cortico-cortical connections. Synaptic loss within these areas or functional dysconnections between state- and policy-representing regions could effectively reduce the policy space (see Friston, Brown, Siemerkus, & Stephan, 2016, for a discussion of the disconnection hypothesis of schizophrenia). It is therefore interesting to note that reductions in frontal and temporal gray matter and reduced synaptic density are features of schizophrenic illness (for a review, see Faludi & Mirnics, 2011).

### Limitations

One limitation of our model is that it assumes a dialogic structure. While this allowed us to produce an agent that can act instead of only perceiving prelearned stimuli, it does not necessarily reflect all cases of AVH—as these do not always have a dialogic component. Real patients can hallucinate in many contexts, but our model produces hallucinations strictly in the context of an ongoing conversation. We acknowledge that our model is not a comprehensive model of communication or language, in that our agent chooses to speak or listen only as a function of the policy she infers and in reaction to her conversational partner’s actions. She does not speak or listen to minimize uncertainty beyond the proprioceptive and auditory domains; that is, the agent does not use language to impart meaning or to satisfy prior preferences (i.e., asking questions to resolve uncertainty). Our agent therefore does not properly employ language but rather a simple form of turn-taking behavior that reflects aspects of language in a rudimentary way. Another limitation is that, for simplicity, we restricted our policy spaces to be oppositional—an agent could only choose to listen or to speak, but not both. However, the purpose of the simulation was to demonstrate the importance of *active* perception, not to comment on specific sequences of inferred actions that may exist in vivo. In addition, we only employed two states, and it is unclear if our findings would generalize to agents with higher dimensional state and policy spaces. Our model simulates the emergence of hallucinations given overly strong priors and a permissively low likelihood precision. While this may be attractive for disorders like schizophrenia, it may be less appropriate for the description of hallucinations in patients losing their sight (Charles Bonnet syndrome) or hearing, where decreased likelihood precision caused by a dysfunctional sensory apparatus allows priors to dominate (without there being any abnormality of the priors), leading to hallucinations (Friston, 2005). As such, this model represents only *one* way in which false positive inferences can be generated.

### Future Directions

In the future, we hope to produce an agent that can modulate its own policy space, perhaps under the influence of affective or memory-related cues from a simulated medial temporal lobe. This would help to explain how an agent might sculpt a hallucinogenic policy space, perhaps when constrained to reduce that space over time to simulate overpruning. We hope to construct models with more complex state spaces to simulate language or conversations. A more complete model may also allow us to investigate the content of hallucinations and how they respond to context. We hope to design tasks that test model predictions that could be completed during fMRI to probe neurobiological correlates.

## CONCLUSION

We have simulated hallucinations using a Markov decision process, under an active inference framework. Hallucinations, defined as false positive inferences, emerged with decreased likelihood precision, combined with a high prior precision over policies or a hallucinogenic policy space. In other words, hallucinations occurred when aberrant but strongly held priors over policies entailed predictions about sensory states that could not be corrected because of imprecise sensory information. This leads to a “precise (prior) belief, imprecise (sensory) evidence” view of AVH. Agents that hallucinate do so because they believe that only certain sequences of events are likely, and they are unable to use sensory information to update these beliefs.

## ACKNOWLEDGMENTS

All simulations were run using the DEM toolbox included in the SPM12 package. This is open-access software provided and maintained by the Wellcome Trust Center for Neuroimaging and can be accessed here: http://www.fil.ion.ucl.ac.uk/spm/software/spm12/.

## AUTHOR CONTRIBUTIONS

David A. Benrimoh: Conceptualization: Lead; Software: Equal; Visualization: Equal; Writing—original draft: Lead; Writing—review & editing: Equal. Thomas Parr: Conceptualization: Equal; Software: Equal; Supervision: Equal; Visualization: Equal; Writing—review & editing: Supporting. Peter Vincent: Conceptualization: Supporting; Writing—review & editing: Supporting. Rick A. Adams: Conceptualization: Supporting; Supervision: Equal; Writing—review & editing: Equal. Karl Friston: Methodology: Lead; Software: Supporting; Supervision: Equal; Writing—review & editing: Supporting.

## FUNDING INFORMATION

DB is supported by a Richard and Edith Strauss Fellowship (McGill University) and the Fonds de Recherche due Quebec–Santé (FRQS). TP is supported by the Rosetrees Trust (Award Number 173346). RAA is supported by the Academy of Medical Sciences (AMS-SGCL13-Adams) and the National Institute of Health Research (CL-2013–18–003). KJF is a Wellcome Principal Research Fellow (Ref: 088130/Z/09/Z).

## Note

^1^ When the outcomes are sensory samples, the likelihood precision plays a role of a sensory precision in predictive coding formulations.
